# Antinociceptive and anti-inflammatory activities of *Geranium bellum* and its isolated compounds

**DOI:** 10.1186/1472-6882-14-506

**Published:** 2014-12-17

**Authors:** Claudia Velázquez-González, Raquel Cariño-Cortés, Juan A Gayosso de Lucio, Mario I Ortiz, Minarda De la O Arciniega, Diana A Altamirano-Báez, Luis Jiménez- Ángeles, Mirandeli Bautista-Ávila

**Affiliations:** Área Académica de Farmacia, Instituto de Ciencias de la Salud, Universidad Autónoma del Estado de Hidalgo, Ex-Hacienda la Concepción, Tilcuautla, Hidalgo México; Área Académica de Medicina, Instituto de Ciencias de la Salud, Universidad Autónoma del Estado de Hidalgo, Ex-Hacienda la Concepción, Tilcuautla, Hidalgo México; Centro Nacional de Investigación en Imagenología e Instrumentación Médica Departamento de Ingeniería Eléctrica, Universidad Autónoma Metropolitana Unidad, Iztapalapa, Ciudad de México México

**Keywords:** *Geranium bellum*, Antinociceptive activity, Anti-inflammatory activity, Geraniin, Quercetin, Ellagic acid

## Abstract

**Background:**

*Geranium bellum* Rose, locally known as “Pata de león”, is a perennial plant distributed in the mountains of Hidalgo, Mexico. It is widely used in Mexican traditional medicine to treat fever, pain, and gastrointestinal disorders. To date, there are not published studies regarding the *in vivo* antinociceptive and anti-inflammatory potential of the acetone-aqueous extract from the aerial parts of *G. bellum*.

**Methods:**

Antinociceptive effects of the acetone-aqueous *G. bellum* (AGB) extract and the isolated compounds were assessed using experimental pain models, including thermal nociception like hot plate test, and chemical nociception induced by intraperitoneal acetic acid or subplantar formalin injection *in vivo*. The anti-inflammatory properties of the extract were studied using systemic administration in carrageenan-induced paw edema.

**Results:**

Intra-gastric administration of AGB (75, 150, and 300 mg/kg) showed a dose-dependent antinociceptive effect in intraperitoneal acetic acid (writhing), thermal nociception in CD1 mice, and subplantar formalin models, as well as anti-inflammatory effect in carrageenan- induced paw edema in Wistar rats. Geraniin and quercetin showed the highest antinociceptive activity in writhing test, whereas ellagic acid was the most active compound in the hot plate model.

**Conclusion:**

These studies provide evidences that *G. bellum* shows antinociceptive and anti- inflammatory effects, and gives support to its use in treating pain in Mexican traditional medicine.

## Background

Pain is a common cause of medical consultation, defined as an unpleasant sensory and emotional experience associated with actual or potential tissue damage [1, 2], it affects all human activities. Drugs to treat pain, highly demanded throughout the world, are usually classified either as opiates (morphine, methadone, or pentazocine) or non- steroidal anti-inflammatory drugs (NSAIDs, acetylsalicylic acid [ASA], diclofenac, indomethacin, or ketorolac). Unfortunately, both opiates and NSAIDs may have adverse reactions such as respiratory depletion, cardiovascular instability, gastric damage, tachycardia, hypotension, hepatotoxicity, nephrotoxicity, Reye’s syndrome, and blood dyscrasia [3]. In recent years, considerable attention has been paid to screening new drugs with analgesic activity from natural sources, to reduce or treat pain with fewer adverse effects than allopathic drugs.

Several studies has been carried out to obtain experimental evidence on the antinociceptive and anti-inflammatory activities of Geranium species and its phenolic compound, the aqueous extract of *G. pratense* subspecie finitimum, showed anti-inflamatory activity on carragenan-induced hind paw edema assay and diminished significantly the number of writhings [4]. *G. nepalense* and isolated compounds showed anti-inflammatory effects on tetradecanoyl phorbol acetate (TPA)-induced mouse ear edema [5]. G. sibiricum regulates the inflammatory reaction stimulated by phorbol-12-myristate 13-acetate plus calcium ionophore A23187 (PMACI) in human mast cells [6].

In this study the antinociceptive and anti-inflamatory activity of *G. bellum* was evaluated. *G. bellum* Rose (Geraniaceae), locally known as “Pata de león”, is a perennial plant distributed in the mountains of Hidalgo, Mexico. It has been used in traditional medicine to treat fever, pain, and gastrointestinal disorders [7]. Phytochemical studies of ethyl acetate extract of aerial parts led to the isolation of β-sitosterol 3-O-β-D-glucopyranoside, quercetin 3-O-α-L-(2”-O-acetyl)- arabinofuranoside, and quercetin 3-O-α-L-arabinofuranoside (avicularin), while from methanol extract of aerial parts led to the isolation of quercetin, methyl gallate, gallic acid, methyl brevifolin carboxylate, dehydrochebulic acid trimethyl ester [8], geraniin, corilagin, gallic acid, quercetin 3-O-β-D-(6”-galloyl)-glucopyranoside, kaempferol, and kaempferol 3-O-β-D-glucopyranoside [9]. Pharmacological studies of isolated compounds has been carried out; methyl-, ethyl-, and butyl-brevifolin carboxylate derivatives inactivated triosephosphate isomerase from *Trypanosoma cruzi* [10], geraniin and ellagic acid showed antitumoral effect [11]. Moreover, ellagic acid showed anti-inflammatory effect on carrageenan-induced paw edema in rats and acute lung injury induced by acid in mice, and analgesic effects on radiant heat tail-flick and acetic acid-induced pain (writhing test) [12–14]. Geraniin and corilagin, exhibited dose-dependent antinociceptive properties in acetic acid-induced pain in mice [15], corilagin presented antiiflammatory activity on the first phase of formalin, glutamate and capsaicin test [16]. Quercetin showed antinociceptive activity in acetic acid-induced pain, inhibited both phases of formalin-induced pain also inhibited the nociception induced by glutamate and capsaicin [17].

Nevertheless, there is not scientific report in the literature on *G. bellum* antinociceptive and anti-inflammatory activities. Therefore, the aim of this study was to examine the possible anti-inflammatory and antinociceptive effects of the extract from *G. bellum* aerial parts and of some isolated compounds, using the hot plate test, chemical nociception induced by intraperitoneal acetic acid injection in CD1 mice, subplantar formalin injection and carrageenan-induced paw edema in Wistar rats.

## Methods

### Extraction

Aerial parts of *G. bellum* were collected from July to August 2009 at the community of Epazoyucan, Hidalgo, Mexico. After proper identification, plant voucher specimens (J.A. Gayosso de Lucio 01) were deposited at the Herbarium of Biological Research Center of the Universidad Autónoma del Estado de Hidalgo. Five-hundred grams of dried and ground plant material were extracted by maceration with 5 L of acetone- water (7:3) at room temperature. After filtration, the crude extract was concentrated to dryness under vacuum, yielding 72 g of crude extract (14.4% yield).

### Compound isolation

**Figure 1:**
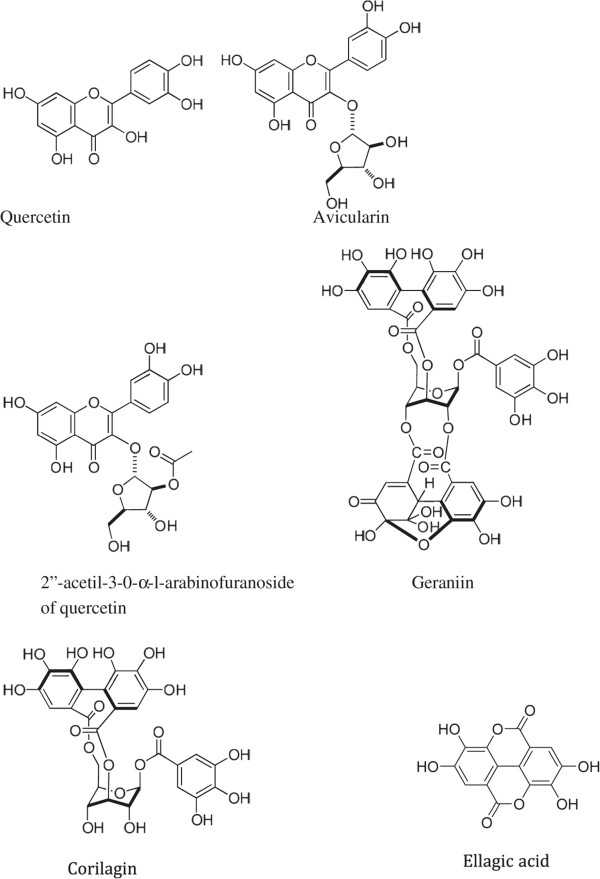
**Isolated compounds from** ***Geranium bellum.***

Two and a half grams of crude extract were fractionated by chromatography on a Sephadex LH-20 column (Pharmacie, Sigma) eluted with H_2_O-MeOH. Eleven fractions of 300 mL were obtained. Fractions 4 and 5 (400 mg) were further purified by two chromatography steps on Merck silica gel (230–400 mesh, ATSM) using CHCl_3_:MeOH:H_2_O in increasing polarity. Fifty fractions of 10 mL were obtained, leading to the isolation of: quercetin, 1 mg (**1**); avicularin, 2.7 mg (**2**); quercetin 3-*O-α*-L-(2”- *O*-acetyl)-arabinofuranoside, 2 mg (**3**). Fractions 6 and 7 (1.5 g) were separated by chromatography on RP18 silica gel using H_2_O:MeOH as eluent. Ten fractions of 5 mL were obtained, leading to the isolation of geraniin, 600 mg (**4**); corilagin, 92 mg (**5**), and ellagic acid, 80 mg (**6**). All isolated compounds were identified by spectroscopic and spectrometric analysis (Figure 1) [9].

### Animals

CD1 female albino mice (30–35 g) and Wistar male rats (150–200 g) were purchased from the vivarium of Universidad Autónoma del Estado de Hidalgo. Animals were kept under standard humidity conditions at 22 ± 2°C under a 12-h light/dark cycle, and fed with a standard diet and allowed water *ad libitum*. Animals were fasted by 12 h prior to the experiments. This study was approved by the Internal Ethics Committee for the Use and Care of Laboratory Animals from Universidad Autónoma del Estado de Hidalgo (CIECUAL) in compliance with Guidelines for Use and Care of Laboratory Animals NOM 0062-ZOO-1999 [18]. Animals were euthanized in a CO_2_ chamber after the experiments.

### Formalin-induced nociception in rats

A formalin solution (1% in 0.9% saline, 50 μl/paw) was injected into the hind paw plantar surface of rats (n = 6) (i.pl.), and the animals were individually placed in transparent observation chambers, as previously described [19, 20]. To assess the systemic antinociceptive effect, oral treatments (p.o.) with the vehicle alone, AGB (75, 150, and 300 mg/kg), diclofenac (30 mg/kg) and indomethacin (30 mg/kg), and were administered 30 min prior to formalin injection. To assess local antinociceptive effect, rats were pretreated (i.pl.) with the vehicle alone, AGB (200, 400, and 800 μg/paw), diclofenac (200 μg/paw) and indomethacin (800 μg/paw) 20 min prior to formalin injection into the ipsilateral paw. The relative time that subject animal spent flinching the injected paw was recorded, and it was expressed as the area under the curve (AUC) of flinch frequency against time.

### Acetic acid-induced writhing in mice

Antinociceptive activity of AGB and isolated compounds was tested using acetic acid-induced writhing [21–23]. Mice (n = 7) were treated with vehicle alone or AGB (75, 150, and 300 mg/kg, p.o.), geraniin, ellagic acid, corilagin, quercetin (5, 10, and 25 mg/kg, p.o.), indomethacin (10 mg/kg, p.o.), or acetylsalicylic acid (200 mg/kg, p.o.) 30 min prior to acetic acid injection. The antinociceptive effect was studied by counting the total number of abdominal contractions (writhing movements) during 25 min after the animal was intraperitoneally injected with 0.1 mL/10 g body weight of 0.6% (v/v) acetic acid solution in distilled water. Antinociceptive activity was expressed as the percentage change in writhing rate with respect to controls and it was expressed as the area under the curve (AUC) of writhing movements against time.

### Hot plate test in mice

Hot plate apparatus (Ugo Basile, Italy) was used to measure nociceptive response [22]. Mice (n = 7) were placed into an acrylic cylinder on the heated surface (55 ± 0.2°C), and the time between the mouse placement on the platform and shaking/licking of the hind paws or jumping was recorded as the response latency. Mice were treated orally with vehicle, geraniin (25 mg/kg), corilagin (10 mg/kg), ellagic acid (10 mg/kg), quercetin (10 mg/kg), or dissolved in 1 ml of 1% Tween 80 in water, p.o., 30 min before thermal noxious stimulus in the hot plate test. Morphine (5 mg/kg) was used as a positive control. Mice were observed before and at 0, 15, 30, 45, 60, 75, 90, 105 and 120 min after drug administration. A cut-off of 30 s was set; this exposition time was enough to observe any animal response without causing tissue damage. Antinociceptive activity was expressed was expressed as area under the curve (AUC) of thermal latency against time.

### Carrageenan-induced rat paw edema

Pedal inflammation in rats was produced, as previously described by Ponce-Monter *et al.* (2010) [24], following an overnight fast with free access to water. Paw edema was measured with a plethysmometer (Model 7140, Comerio, Italy). The basal volume of the right hind paw was determined before any drug administration. After basal volume was determined, animals (n = 6) were divided into experimental groups in such a way that the mean volumes were similar among groups. Vehicle, AGB (75, 150, and 300 mg/kg), diclofenac (30 mg/kg), and indomethacin (30 mg/kg) were orally administrated 30 min before i.pl. injection of carrageenan (100 μl/paw). Paw volume was measured 6 h after the inflammatory stimulus. Data were expressed as percent of the anti-inflammatory effect.

### Statistical analysis

All experimental results are reported as mean ± S.E.M. for 6–8 animals per group. The area under latency-time curves (AUC), expressing the effect duration, was calculated by the trapezoidal rule [25]. One-way analysis of variance (ANOVA) followed by Tukey’s test was used to compare differences between treatments. Differences were considered as statistically significant when *P* < 0.05.

## Results

In the present study we evaluated the antinociceptive and anti-inflammatory activities of AGB, geraniin, corilagin, ellagic acid and quercetin, by using formalin-induced nociception, acetic acid-induced writhing, hot plate test, and carrageenan-induced rat paw edema tests.

### Formalin-induced nociception in rats

**Figure 2:**
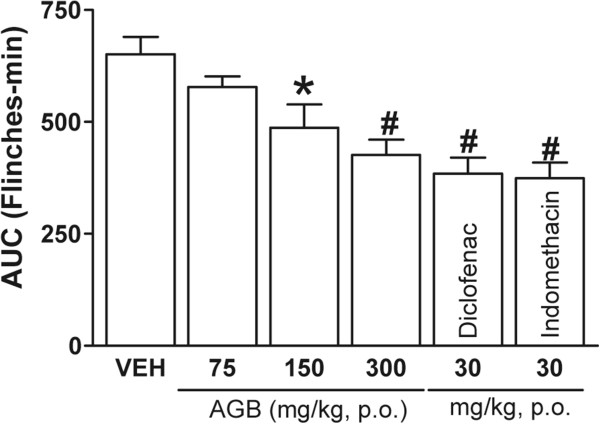
**Systemic antinociceptive effect of acetone-aqueous extract of** ***G. bellum*** **(AGB), diclofenac, and indomethacin on the 1%** **formalin test.** Prior to formalin injection, rats (n = 6) were systemically administered with vehicle (VEH), AGB, diclofenac, or indomethacin. Data are expressed as the area under the curve (AUC) of the number of flinches against time. Significantly different from vehicle group (*P < 0.05 and #*P* < 0.01), as determined by analysis of variance followed by Tukey’s test.

**Figure 3:**
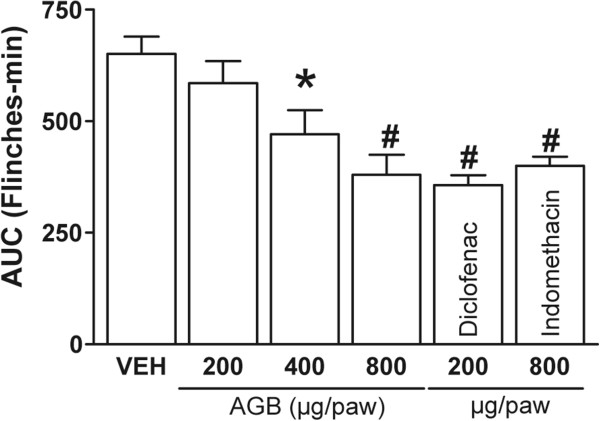
**Local antinociceptive effect of acetone-aqueous extract from** ***G. bellum*** **(AGB), diclofenac, and indomethacin in 1%** **formalin test.** Rats (n = 6) were pretreated with a s.c. injection of vehicle, AGB, diclofenac, or indomethacin into either paw before formalin injection. Data are expressed as the area under curve (AUC) of the number of flinches against time. Significantly different from vehicle group (*P < 0.05 and #*P* < 0.01), as determined by analysis of variance followed by Tukey’s test.

Systemic (150 and 300 mg/kg) and local peripheral (400 and 800 μg/paw) AGB significantly inhibited formalin-induced nociception in rats (Figures 2 and 3) observed as AUC (*P* < 0.05 and *P* < 0.01). Inhibition rates of formalin-induced flinching compared with the vehicle group were 34.5% at 300 mg/kg and 41.7% at 800 μg/paw in systemic and local peripheral tests, respectively. Inhibitory effects in the measured time of formalin-induced flinches by AGB were similar to positive controls, indomethacin and diclofenac (*P* < 0.01) at the highest dose in both tests.

### Acetic acid-induced writhing in mice

**Figure 4:**
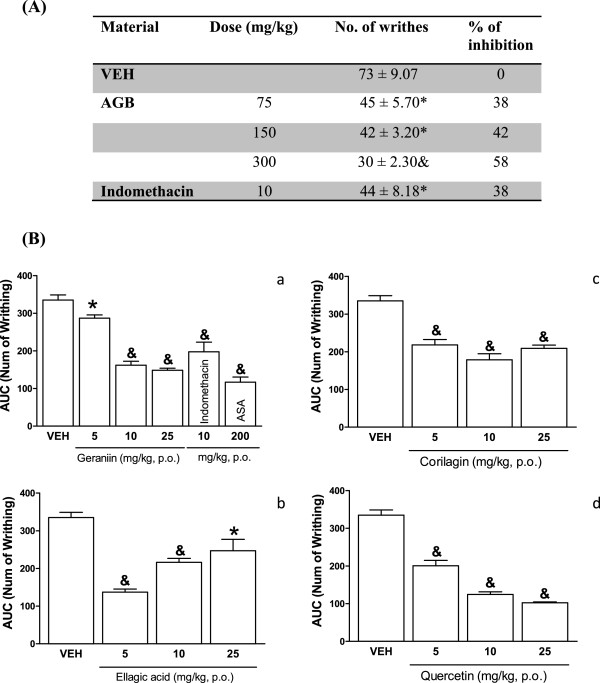
**Systemic antinociceptive effect of acetone-aqueous extract of from** ***G. bellum*** **(AGB), (panel A) and pure compounds (panel B) in writhing test.** Mice (n = 7) were orally administered either with vehicle (VEH), AGB, geraniin, ellagic acid, corilagin, quercetin, acetylsalicylic acid (ASA) or indomethacin 30 min before test. The number of writhes was counted over a 30 min period following the injection of 1% acetic acid. Data are expressed as the percent of the anti-inflammatory effect (A) and the area under curve (AUC) of the number of writning by 25 min. Significantly different from the vehicle group (*P < 0.05 and &*P* < 0.001) as determined by the analysis of variance followed by Tukey’s test.

Cumulative frequency of abdominal stretching correlated with the level of acetic acid- induced pain (Figure 4). Oral AGB administration (75, 150, and 300 mg/kg) reduced the number of acetic acid-induced abdominal constrictions in comparison with the vehicle group AGB (Figure 4A). Geraniin, corilagin, ellagic acid, and quercetin (5, 10, and 25 mg/kg) (Figure 4B) observed as AUC (*P* < 0.05 and *P* < 0.001) showed similar or greater inhibition rates of writhing frequency than the positive control (indomethacin, 10 mg/kg).

### Hot plate test in mice

**Figure 5:**
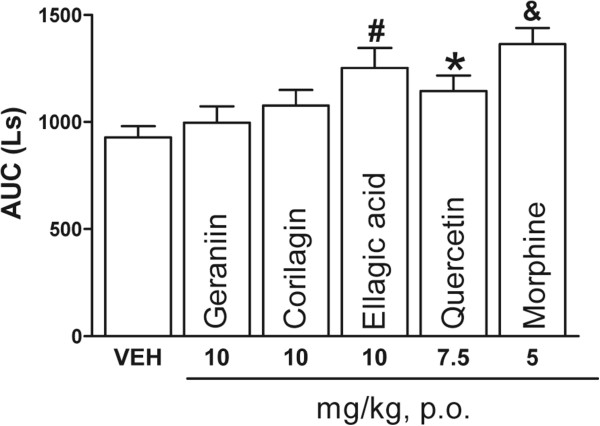
**Antinociceptive effect of geraniin, corilagin, ellagic acid (10 mg/kg), quercetin (7.5 mg/kg), and morphine (5 mg/kg).** Data in the hot plate test consist of the area under the curve (AUC) of thermal latency against time in mice (n = 7). Thermal latency was assessed during 2 h. Significantly different from vehicle group (*P < 0.05, #*P* < 0.01 and &*P* < 0.001), as determined by analysis of variance followed by Tukey’s test.

On the hot plate assay, only ellagic acid (10 mg/kg) and quercetin (7.5 mg/kg) significantly increased the latency to thermal stimulus observed as AUC (*P* < 0.05 and *P* < 0.01, respectively) (Figure 5). The observed pharmacological action was similar than morphine (5 mg/kg). However, no increase in latency to thermal stimulus was observed either with geraniin or corilagin (10 mg/kg).

### Carrageenan-induced rat paw edema

**Figure 6:**
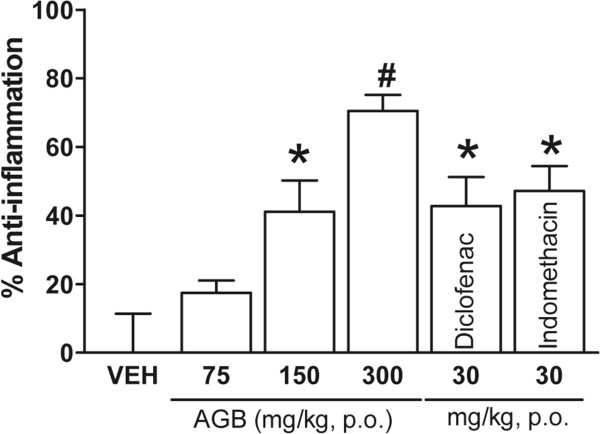
**Systemic anti-inflammatory effect of acetone-aqueous extract of** ***G. bellum*** **(AGB) (75, 150 and 300 mg/kg), diclofenac (30 mg/kg), and indomethacin (30 mg/kg) in carrageenan test.** Solutions were orally administrated 30 min before carrageenan i.pl. injection (100 μL/paw) in rats (n = 6). Paw volume was measured 6 h after the inflammatory stimulus. Data are expressed as the percent of the anti-inflammatory effect. Significantly different from the vehicle group (*P < 0.05 and #*P* < 0.01) as determined by analysis of variance followed by Tukey’s test.

AGB significantly inhibited carrageenan-induced rat paw edema (Figure 6) at doses of 150 and 300 mg/kg (*P* < 0.05 and *P* < 0.01, respectively), 6 h after carrageenan administration. Edema inhibition rates were 41.1% and 70.5% for 150 and 300 mg/kg of AGB, respectively. Indomethacin and diclofenac (30 mg/kg) yielded an inhibition rate of 42.8% and 47.2%, respectively.

## Discussion and conclusions

*G. bellum* is widely used in folk medicine at the mountains of Hidalgo State, Mexico to treat fever, pain, and gastrointestinal disorders [7], however there is not report on the antinociceptive activity of this plant in the scientific literature. Results obtained in this study showed antinociceptive and anti-inflammatory activity of AGB and isolated compounds from *G. bellum* Rose in classical pharmacological models of pain.

To distinguish between AGB central and peripheral antinociceptive action, the formalin test was performed. This test, believed to represent an appropriate model of clinical pain, involves two distinct phases. The first phase, neurogenic pain, occurs approximately 3 min after formalin injection. After a quiescent period, a second phase, inflammatory pain, occurs between 20 and 30 minutes post-injection. Phase 1 result from the direct stimulation of nociceptors, whereas phase 2 involves a period of sensitization during which inflammatory phenomena occur. In this experiment, AGB showed both systemic and local antinociceptive effects, decreasing the flinching behavior during phase 2. A decrease in flinching time in both phases is characteristic of centrally acting drugs, and points to a possible interaction with opioid receptors [19, 26, 27]. Opioid analgesics seem to be antinociceptive in both phases, although phase 1 is more sensitive to these substances. In contrast, NSAIDs such as indomethacin seem to suppress only phase 2, while the analgesic effect of diclofenac involves not only its anti-inflammatory action, since its peripheral antinociceptive effect is associated with ATP-sensitive K^+^ channels [28]. AGB local and systemic effects in phase 2 indicate a possible anti-inflammatory effect, inhibiting the release of inflammatory mediators (arachidonic acid metabolites) that sensitize and activate peripheral nociceptors.

The nociceptive response caused by acetic acid is also dependent on the release of some cytokines, such as TNF-α and interleukin-1 and 8, by modulating the response of macrophages and mast cells located in the peritoneal cavity [29, 30]. In all tested doses, AGB showed moderate antinociceptive activity in a dose-dependent way, at 75 mg/kg, it possesses antinociceptive activity similar to indomethacin (10 mg/kg) (Figure 4A), whereas at 300 mg/kg it was better than the control drug. The results of this study demonstrate that antinociceptive activity of all evaluated compounds in the writhing test are similar to that of indomethacin, being significant the activity of geraniin (10 and 25 mg/kg) and quercetin (5, 10, and 25 mg/kg), with respect to the vehicle. This results are according to previous studies where geraniin and corilagin, exhibited dose-dependent antinociceptive properties in acetic acid-induced pain in mice [15].

The antinociceptive effect of ellagic acid and geraniin was demonstrated on acetic acid- induced abdominal contractions in mice [12, 15]. These results are comparable with our study on female CD1 albino mice. The mechanism of action of AGB and its metabolites could be partly linked to the lipoxygenase and/or cyclooxygenase system, taking into account that acetic acid increases prostaglandin levels (PGE_2_ and PGF_2_) in peritoneal fluid [31–33].

To corroborate the participation of the central analgesic system in the antinociceptive activity of our pure compounds, the hot plate test was employed. Hot plate test is a nociception model based on a high-intensity phasic stimulus. It is a central model that shows selectivity for opioid-derived analgesics, such as morphine. In the hot plate test, ellagic acid showed a similar activity as morphine, quercetin also exhibited significant effect, while geraniin and corilagin did not show any significant effect in this model. These results suggest that ellagic acid and quercetin could act in the central analgesic system, due some studies have reported participation of the opioidergic for quercetin [34].

In carrageenan-induced rat paw edema test, inflammatory response induced by carrageenan is characterized by a biphasic response, marked edema resulting from the rapid production of several inflammatory mediators such as histamine, serotonin, and bradykinin is observed in the first phase. The second phase is characterized by the release of prostaglandins and nitric oxide produced by inducible isoforms of COX (COX-2) and nitric oxide synthase (iNOS), respectively, reaching a peak at 3 h [35]. In this study, the anti-inflammatory activity of AGB was evaluated using the carrageenan-induced rat paw edema test. Oral administration of AGB suppressed the edematous response in a dose-dependent manner 6 h after carrageenan injection. The inhibitory effect in this model may be due to the inhibition of cyclooxygenase, since its effect can be compared to that caused by indomethacin; it is noteworthy that at 150 mg/kg there was no difference with the controls, whereas at 300 mg/kg the anti-inflammatory activity was even better than indomethacin and diclofenac. Flavonoids have been reported as good antioxidant and antinociceptive agents, and they have been shown to inhibit cyclooxygenase, lipoxygenase, microsomal monooxygenase, glutathione S-transferase, mitochondrial succinoxidase, and NADPH-oxidase, all enzymes involved in generating reactive oxygen species. They also possess antioxidant activity by inhibiting COX-2, tyrosine and threonine kinase, phosphatidylinositol-3-kinase (PI_3_Q), and phosphatidylinositol-5-kinase (PI_5_Q), enzymes related to anti-inflammatory process and directly or indirectly to pain signaling mechanisms [36]. It was reported that glycoside derivatives of quercetin isolated from *G. pratenses* showed anti-inflammatory and antinociceptive activity [4]. In conclusion, the results presented in this study suggest that aerial parts of *G. Bellum* Rose, as well as the pure compounds isolated from them, possess anti-inflammatory and antinociceptive peripheral activity when locally and systemically administered, while ellagic and quercetin also showed thermal-induced antinociception. The pure compounds showing antinociceptive activity might act synergistically or individually to contribute to the analgesic activity of the plant, and suggest that *G. bellum* may be a good candidate for the treatment of mild pain. This study gives support to the use of this plant in traditional medicine to treat pain.

## References

[1] IASP (1979). Subcommittee on Taxonomy Pain terms: a list with definitions and notes on usage. Recommended by the IASP Subcommittee on Taxonomy. Pain.

[2] Puebla D (2005). Tipos de dolor, escala terapéutica de la OMS dolor iatrogénico. Oncología (Barc).

[3] Lacy F, Armstrong L, Goldman M, Lance L (2008). Drug Information Handbook.

[4] Küpeli E, Tatli II, Akdemir ZS, Yesilade E (2007). Estimation of antinociceptive and antiinflammatory activity of *Geranium pretense* subsp finitum and its phenolic compounds. J Ethnopharmacol.

[5] Lu CH, Li YY, Li LJ, Liang LY, Shen YM (2012). Anti-inflammatory activities of fractions from *Geranium nepalense* and related polyphenols. Drug Discov Ther.

[6] Shim JU, Oh PS, Lim KT (2009). Anti-inflammatory activity of ethanol extract from *Geranium sibiricum* Linne. J Ethnopharmacol.

[7] Pérez-Escandón BE, Villavicencio MA (1995). Listado de las Plantas Medicinales del Estado de Hidalgo.

[8] Camacho A, Gayosso JA, Torres JM, Muñoz JL, Alarcón E, López R, Barrón BL (2008). Antioxidant Constituents of *Geranium bellum* Rose. J Mex Chem Soc.

[9] Gayosso JA, Torres JM, Cerda CM, Nathan J (2010). Ellagitannins from *Geranium potentillaefolium* and *G. bellum*. Nat Prod Commun.

[10] Gayosso JA, Torres JM, Rojo A, Najéra H, Aguirre B, Salas J, Avitia C, Tellez A (2009). Selective inactivation of triosephosphateisomerase from *Trypanosoma cruzi* by brevifolincarboxylate derivatives isolated from *Geranium bellum* Rose. Bioorg Med Chem Lett.

[11] Kashiwada Y, Nonaka G, Nishioka I, Chang JJ, Lee KH (1992). Antitumor agents, 129, Tannins and related compounds as selective cytotoxic agents. J Nat Prod.

[12] Rogerio AP, Fontanari C, Melo MC, Ambrosio SR, de Souza EG, Pereira PS, França CS, da Costa FB, Albuquerque DA, Faccioli LH (2006). Anti-inflammatory, analgesic and anti-oedematous effects of *Lafoensia pacari* extract and ellagic acid. J Pharm Pharmacol.

[13] Mansouri T, Naghizadeh B, Ghorbanzadeh B, Farbood Y (2013). Central and peripheral antinociceptive effects of ellagic acid in different animal models of pain. Eur J Pharmacol.

[14] Favarin DC, Teixeira MM, Lemos de Andrade E, De Feitas C, Lazo JE, Sorgi AC, Faccioli LH, Rogerio AP (2013). Anti-inflammatory effects of ellagic acido on acutte lung injury induced by acid in mice. Mediators Inflamm.

[15] Miguel OG, Calixto JB, Santos AR, Messana I, Ferrari F, Cechinel Filho V, Pizzolatti MG, Yunes RA (1996). Chemical and preliminary analgesic evaluation of geraniin and furosin isolated from *Phyllanthus sellowianus*. Planta Med.

[16] Moreira J, Klein-Júnior LC, Cechinel Filho V, de Campos BF (2013). Anti-hyperalgesic activity of corilagin, a tannin isolated from *Phyllanthus niruri* L. (Euphorbiaceae). J Ethnopharmacol.

[17] Filho AW, Filho VC, Olinger L, de Souza MM (2008). Quercetin: further investigation of its antinociceptive properties and mechanisms of action. Arch Pharm Res.

[18] NOM (1999). Mexican Official Norm for Animal Care and Handing (NOM-062- ZOO-1999).

[19] Ortiz MI, Castañeda HG (2008). Examination of the interaction between peripheral lumiracoxib and opioids on the 1% formalin test in rats. Eur J Pain.

[20] Ortiz MI (2012). Metformin and phenformin block the peripheral antinociception induced by diclofenac and indomethacin on the formalin test. Life Sci.

[21] Koster R, Anderson M, DeBeer EJ (1959). Acetic acid for analgesic screening. J Fed Proc.

[22] Cariño CR, Gayosso JA, Ortíz MI, Sánchez M, García PB, Cilia VG, Pérez N, Moreno E, Ponce MH (2010). Antinociceptive, genotoxic and histopathological study of Heliopsis longipes S.F. Blake in mice. J Ethnopharmacol.

[23] Cássia R, Gomes L, Ferreira F, Bhattacharyya J, Nóbrega R (2010). Antinociceptive and toxicological effects of *Dioclea gradiflora* Seed Pod in Mice. J Biochem Biophys Methods.

[24] Ponce MH, Fernández ME, Ortiz MI, Ramírez MML, Cruz ED, Pérez HN, Cariño CR (2010). Spasmolytic and anti-inflammatory effects of *Aloysia triphylla* and citral, *in vitro* and *in vivo* studies. J Smooth Muscle Res.

[25] Tallarida RJ, Murray RB (1986). Manual of Pharmacologic Calculations with Computer Programs.

[26] Dubuisson D, Dennis SG (1977). The formalin test: a quantitative study of the analgesic effects of morphine, meperidine, and brain stem stimulation in rats and cats. Pain.

[27] Malmberg AB, Yaksh TL (1992). Antinociceptive actions of spinal nonsteroidal anti- inflammatory agents on the formalin test in the rat. J Pharmacol Exp Ther.

[28] Ortiz MI, Castañeda HG, Izquierdo VJA, Sánchez GM, Ponce MHA, Granados SV (2012). Role of ATP-sensitive K^+^ channels in the antinociception induced by non-steroidal anti-inflammatory drugs in streptozotocin-diabetic and non-diabetic rats. Pharmacol Biochem Behav.

[29] Ribeiro RA, Vale ML, Ferreira SH, Cunha FQ (2000). Analgesic effect of thalidomide on inflammatory pain. Eur J Pharmacol.

[30] Couture R, Harrisson M, Vianna RM, Cloutier F (2001). Kinin receptors in pain inflammation. Eur J Pharmacol.

[31] Ojewole JA (2006). Antinociceptive, anti-inflammatory antidiabetic properties of Hypoxis hemerocallidea Fisch. and C.A. Mey (Hypoxidaceae) corm ‘African potato’ aqueous extract in mice and rats. J Ethnopharmacol.

[32] Dou W, Jiao Y, Goorha S, Raghow R, Ballou LR (2004). Nociception and the differential expression of cyclooxygenase-1 (COX-1), the COX-1 variant retaining intron-1 (COX-1v) and COX-2 in mouse dorsal root ganglia (DRG). Prostaglandins Other Lipid Mediat.

[33] Ballou LR, Botting RM, Goorha S, Zhang J, Vane JR (2000). Nociception in cyclooxygenase isozyme-deficient mice. Proc Natl Acad Sci U S A.

[34] Martínez AL, González-Trujano ME, Aguirre-Hernández E, Moreno J, Soto-Hernández M, López-Muñoz FJ (2009). Antinociceptive activity of Tilia americana var. mexicana inflorescences and quercetin in the formalin test and in an arthritic pain model in rats. Neuropharmacology.

[35] Seibert K, Zhang Y, Leahy K, Hauser S, Masferrer J, Perkins W, Lee L, Isakson P (1994). Pharmacological and biochemical demonstration of the role of cyclooxygenase 2 in inflammation and pain. Proc Natl Acad Sci U S A.

[36] Moreira AS, Spitzer V, Schapoval EE, Schenkel EP (2000). Antiinflammatory activity of extracts and fractions from leaves of *Gochanatia polymorpha*. Phytother Res.

[CR37] The pre-publication history for this paper can be accessed here:http://www.biomedcentral.com/1472-6882/14/506/prepub

